# Surveillance of Influenza Viruses in Waterfowl Used As Decoys in Andalusia, Spain

**DOI:** 10.1371/journal.pone.0098890

**Published:** 2014-06-05

**Authors:** Estefanía Jurado-Tarifa, Sebastian Napp, Juan Manuel Gómez-Pacheco, Manuel Fernández-Morente, Juan Antonio Jaén-Téllez, Antonio Arenas, Ignacio García-Bocanegra

**Affiliations:** 1 Departamento de Sanidad Animal, Facultad de Veterinaria, Universidad de Córdoba-Agrifood Excellence International Campus, Córdoba, Spain; 2 Centre de Recerca en Sanitat Animal (CReSA), Universitat Autònoma de Barcelona-Institut de Recerca i Tecnología Agralimentàries (UAB-IRTA), Bellaterra, Barcelona, Spain; 3 Laboratorio de Sanidad y Producción Animal, Consejería de Agricultura, Pesca y Desarrollo Rural de la Junta de Andalucía, Córdoba, Spain; 4 Servicio de Sanidad Animal, Consejería de Agricultura, Pesca y Desarrollo Rural de la Junta de Andalucía, Sevilla, Spain; 5 Agencia de Gestión Agraria y Pesquera de Andalucía, Consejería de Agricultura, Pesca y Desarrollo Rural de la Junta de Andalucía, Sevilla, Spain; University of Liverpool, United Kingdom

## Abstract

A longitudinal study was carried out to determine the seroprevalence of avian influenza viruses (AIVs) in waterfowl used as decoys in Andalusia, southern Spain. A total of 2319 aquatic birds from 193 flocks were analyzed before and after the hunting season 2011–2012. In the first sampling, 403 out of 2319 (18.0%, CI_95%_: 15.8–19.0) decoys showed antibodies against AIVs by ELISA. The AI seroprevalence was significantly higher in geese (21.0%) than in ducks (11.7%) (*P*<0.001). Besides, the spatial distribution of AIVs was not homogeneous as significant differences among regions were observed. The prevalence of antibodies against AIVs subtypes H5 and H7 were 1.1% and 0.3%, respectively, using hemagglutination inhibition test (HI). The overall and H5 seroprevalences slightly increased after the hunting period (to 19.2% and 1.4%, respectively), while the H7 seroprevalence remained at the same level (0.3%). The proportion of flocks infected by AIVs was 65.3%, while 11.2% and 4.9% of flocks were positive for H5 and H7, respectively. Viral shedding was not detected in any of the 47 samples positive by both ELISA and HI, tested by RRT-PCR. The individual incidence after the hunting season was 3.4%. The fact that 57 animals seroconverted, 15 of which were confirmed by HI (12 H5 and 3 H7), was indication of contact with AIVs during the hunting period. The results indicate that waterfowl used as decoys are frequently exposed to AIVs and may be potentially useful as sentinels for AIVs monitoring. The seroprevalence detected and the seropositivity against AIVs H5 and H7, suggest that decoys can act as reservoirs of AIVs, which may be of animal and public health concern.

## Introduction

Avian influenza viruses (AIVs) are among the most important emerging zoonotic pathogens worldwide, affecting a wide variety of avian and mammalian species, including humans [1]. Most strains of AIVs are low pathogenic (LPAIVs), causing minimal disease in infected animals. However, the H5 and H7 subtypes have implications for public and animal health owing to their potential to mutate to highly pathogenic viruses (HPAIVs), inducing severe disease and high mortality [2]. Public health relevance of AIVs is highlighted by the fact that the H5N1 subtype has caused, up to January 2014, 650 human cases, of which 386 died [3]. It emerged in Southern China in the 1990s, but it was not until the winter of 2005/2006 that spread westward, mainly via migratory birds, reaching Central Asia, Europe and Africa [4]. In March 2013, a novel reassortant AIV (H7N9) was identified in China [5], and has caused, up to January 2014, 251 human cases with 56 deaths [3]. Whether this variant may reach the wild bird population in Europe is difficult to predict, so in this context it is essential to maintain the European Union wild bird surveillance [6]. Wild aquatic birds, especially Charadriiformes and Anseriformes, are considered natural reservoirs of AIVs, and do not usually develop clinical signs [2], [7]. Waterfowl can play an important role in the transmission of LPAI and HPAI strains to poultry farms through long distances during migrations [8]. Besides, AIVs may also persist in the environment for long periods under appropriate conditions, favoring its transmission and maintenance between wild and domestic birds [9].

Due to the strategic location on the migratory flyway of wild birds between Eurasia and Africa, the high number of wetlands and the diversity of wild bird species, Spain is considered a risk area for HPAIVs introduction [10]. Since 2004, a National Avian Influenza Surveillance program has been implemented to determine the incidence of H5 and H7 subtypes of AIVs in wild and domestic birds in Spain. To date, four outbreaks of AIVs have been reported in this country. In 2006, H5N1 strain of HPAIV was detected in a Great Crested Grebe (*Podiceps cristatus*) found dead in the Basque Country (Northern of Spain) [11]. Two outbreaks were detected in poultry in 2009, one of an H5N3 strain of LPAIV in a duck meat production farm in the Community of Navarra (Northern Spain) [12], and the second one associated to an H7N7 strain of HPAIV in a laying hen farm in Guadalajara (Central Spain), next to a wetland with high density of wild birds [13]. In May 2013, an H7N1 strain of LPAI induced illness and mortality in a breeding hen farm in Catalonia, Northern Spain [14]. Although no circulation of HPAI has been detected in Andalusia, this area is a usual route of different migratory species between Europe and Africa. Moreover, previous studies have determined the presence of antibodies against different subtypes of AIVs in resident and migratory waterfowl species in Andalusia [15], [16].

Small game is considered an important economic activity in Spain. Waterfowl are used as decoys and present great relevance in certain regions with presence of wetlands. Decoys are domestic waterfowl species, including Anseriformes and Charadriiformes, reared by hunters in their backyards, which act as lures for hunting purposes (Decree 182/2005, 26 July). The use of decoys in hunting results in frequent contact with wild birds, and therefore a high risk of diseases transmission, not only from wild bird to decoys but also from decoys to human [17]. In this sense, the potential role of hand-reared ducks in AIVs transmission, given the risk of viral exchange between game bird facilities and wild habitats, has been previously suggested [18], [19].

The aim of the present study was to determine the prevalence and incidence of AIVs in aquatic birds used as decoys in Andalusia, before and after the hunting period 2010–2011, and to detect circulation of H5 and H7 subtypes in their populations.

## Materials and Methods

### Ethical Statement

This study complies with the current laws regarding ethics and animal use for scientific purposes in Andalusia where samples were gathered. All necessary licenses were obtained for this study. Permits for the captures and blood collections of decoys were provided by the Regional Government of Andalusia, Spain (Register number: BOJA-246/2010). No ethical approval was deemed necessary as this study did not involve killing of animals. Data and samples were obtained by official veterinarians from Consejería de Agricultura, Pesca y Desarrollo Rural of the Regional Government of Andalusia, and handling procedures and sampling frequency were designed to reduce stress and health risks for subjects, according to European (86/609) and Spanish laws (R.D. 223/1988, R.D.1021/2005).

### Study Design

**Figure 1:**
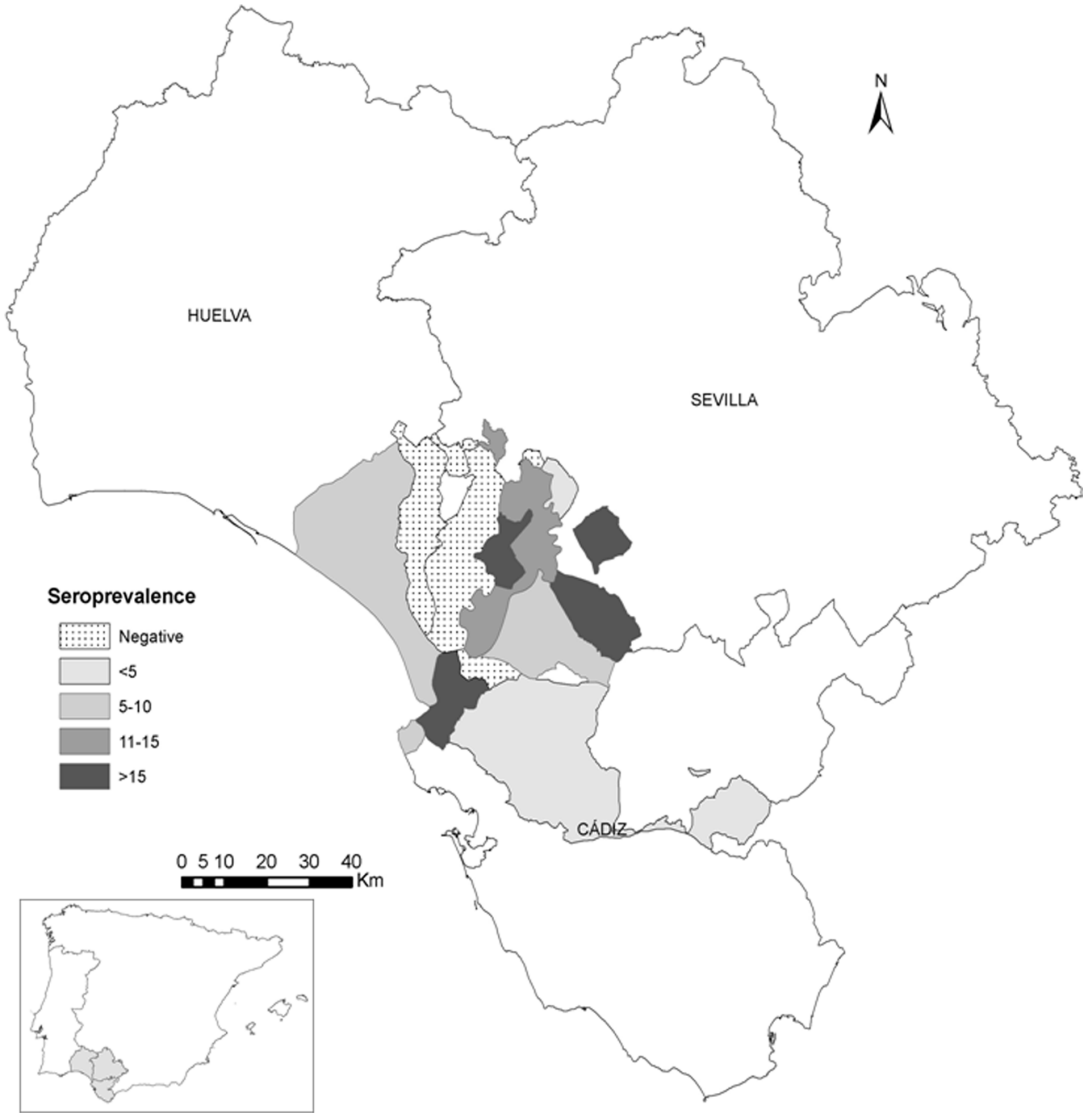
Map of Andalusia (Southern Spain) showing the location of decoys sampled. The darker gray gradient represents the seroprevalence against AIVs in the different municipalities sampled.

The longitudinal study was conducted in Andalusia, located in southern Spain (36°N – 38° 60′ N, 1° 75′ W – 7° 25′ W) (Figure 1). Andalusia is characterised by a Continental thermo Mediterranean climate with hot, dry summers and mild winters. A surveillance program was launched by the Regional Government of Andalusia in December 2010 in order to detect circulation of AIVs in waterfowl species used as decoys in that region [20]. The total census of decoys (n = 2319) was analyzed before the hunting season 2011–2012 (between November and December 2011). Besides, 2110 out of those animals were re-sampled after the hunting season (between February and March 2012). A total of 143 flocks, with sizes ranging from 4 to 29 animals (median = 17), were sampled. The birds tested included 753 ducks, 1557 geese and 9 unidentified birds. All birds were individually identified by metal rings and bled using syringes from the brachial or femoral vein. Blood samples were collected into sterile tubes without anticoagulant and centrifuged at 400 *g* for 15 min. Then, serum was separated and stored at −20°C until analyzed. Moreover, data on sampling date, flock location, census, individual identification and species (ducks or geese) were recorded.

### Serological Analysis

The presence of antibodies against the nucleoprotein of AIVs type A was determined using a commercial blocked enzyme linked assay (ELISA) (10.FLU.K.3. INGEZIM INFLUENZA A, Ingenasa, Madrid, Spain) according to the manufacturer’s recommendations. Sera positive against AIVs type A were then analyzed by means hemagglutination inhibition test (HI) as previously described [21]. Sera that showed HI titers ≥1∶16 were considered as positive. ELISA positive samples were tested for the detection of specific antibodies against H5 using inactivated antigens of both H5N3 (A/Teal/Eng/7394-2805/06) and H5N1 (A/Ck/Scot/59) strains. A serum was considered positive to H5 subtype when showed positive results against both H5N3 and H5N1. ELISA positive samples were also tested for the detection of specific antibodies against H7 using inactivated antigens of both H7N1 (A/Afr.Star/Eng/983/79) and H7N7 (A/Tky/Eng/647/77) strains. A serum was considered positive to H7 subtype when showed positive results against both H7N1 and H7N7. The strains used in the present study were provided by the European AIVs Reference Laboratory in Weybridge (United Kingdom). Hemagglutination inhibition tests were performed at the National AIVs Reference Laboratory in Algete (Madrid).

### Virological Analysis

After serological results, a convenience sampling was carried out to detect AIVs shedding from birds positive by ELISA. A total of 47 cloacal swabs (34 from the first sampling and 13 from the second), from 44 selected seropositive birds by ELISA were collected. Nine of the 44 seropositive birds were also positive against H5 by HI test. Samples were analyzed using an one-step real time reverse transcription polymerase chain reaction (RRT-PCR) with Taqman specific for the matrix gen (gene M) in the segment 7 of AIVs using primers previously described [22]. Briefly, viral RNA was extracted by using BioSprint 96 DNA Blood Kit and poly A RNA carrier (Qiagen, Valencia, CA) following the manufacturer’s instructions. Amplification was performed using a one-step RT-PCR in ABI 7300 equipment (Applied Biosystems, Foster City, CA).

### Statistical Analysis

The prevalence of antibodies against AIVs was estimated from the ratio of positive to the total number of samples, with the exact binomial confidence intervals of 95% [23]. Associations between the serological results and explanatory variables (municipality, region, species, census) were analyzed using a Generalized linear mixed-effects models with an underlying binomial distribution (log link). Models were fitted by Laplace approximation, implemented in the glmer function of the lme4 package for R (http://CRAN.R-project.org/package=lme4). Inference was based on model comparison of nested models (ANOVA), and the process of model selection was based on the lowest Akaike information criterion (AIC) value. Statistical analyzes were carried out in R software (http://www.r-project.org/).

## Results

Antibodies against AIVs were found in 418 out of 2319 (18.0%, CI_95%_: 15.9–19.0) birds tested by ELISA during the first sampling (Table 1). The effect of clustering of animals within flocks was assessed by comparing the model with no explanatory variables (Table 2), but with flocks included as random effect (Model 1), with the model with no explanatory variables, and no random effects (Model 0). The lower value of AIC for Model 1 compared to Model 0, indicates that is important to account for the fact that animals are clustered within flocks. The model with the lowest AIC, and therefore selected (Model 3) included flocks as random effect, and species and provinces as fixed effects. There were differences in seropositivity between geese (21.0%; 327/1557) and ducks (11.7%; 88/753). The results of the model (Table 2) indicate that seropositivity was significantly higher in geese as compared to ducks (OR = 2.5; 95%CI = 1.8–3.4). On the other hand, there were also differences in seropositivity among provinces: 6.1% in Huelva, 16.9% in Cadiz and 20.0% in Seville (Figure 1). The results of the model (Table 2) indicate that seropositivity was significantly higher in Cadiz (OR = 5.7; 95%CI = 1.4–25.3) and in Seville (OR = 8.4; 95%CI = 2.3–35.1) as compared to Huelva.

**Table 1:** Seroprevalences against AIVs in decoys in Andalusia (southern Spain) before and after the hunting season 2010–2011.

Seroprevalence		% positive ELISA (number/overal)	% positive H5 (number/overal)	% positive H7 (number/overal)
Individual	First sampling	*18.0 (418/2319)*	*1.1(25/2279)*	*0.3 (7/2304)*
	Second sampling	*19.2 (406/2110)*	*1.4 (28/2006)*	*0.3 (7/2006)*
Flock	First sampling	*65.7 (94/143)*	*11.2 (16/143)*	*4.9 (7/143)*
	Second sampling	*65.2 (88/135)*	*11.8 (16/135)*	*4.4 (6/135)*

**Table 2:** Results of model selection process.

	*Model 0*	*Model 1*	*Model 2*	*Model 3*
	*No random effects*	*Random effects*	*Random effects*	*Random effects*
	*No fixed effects*	*No fixed effects*	*Fixed effect: species*	*Fixed effects: species+provinces*
**AIC**	***2187***	***1908***	***1873***	***1866***
**Random effects (Flock)**				
Variance		*2.2*	*2.3*	*2.1*
**Fixed effects**				
Intercept		*−2.2*	*−2.8*	*−4.7*
* Geese* † *OR (95%CI)*			*2.5 (1.8–3.4)*	*2.5 (1.8–3.4)*
* Cadiz* ‡ *OR (95%CI)*				*5.7 (1.4–25.3)*
* Seville* ‡ *OR (95%CI)*				*8.4 (2.3–35.1)*

† Ducks as the reference category;

‡ Huelva as the reference category.

A total of 403 seropositive animals could be analyzed by the HI test (15 of the 418 samples had insufficient volume). The individual seroprevalence against H5 and H7 subtypes were 1.1% (25/2279; CI_95%_: 0.7–1.6) and 0.3% (8/2304; CI_95%_: 0.2–0.7), respectively (Table 1).

Ninety four out of 143 flocks (65.7%) had at least one positive animal by ELISA (Table 1). The seroprevalence within positive flocks ranged from 4.2 to 90.5% (mean = 25.3). Antibodies against H5 were detected in 16 of the 143 flocks (i.e. prevalence of 11.2%), while antibodies against H7 were detected in 7 of the 143 flocks (i.e. prevalence of 5.9%). The within flock seroprevalence ranged between 5.9% and 57.1% for H5, and between 5.9% and 33.3% for H7.

The individual seroprevalence increased slightly after the hunting season (to 19.2%; 406/2110). A total of 209 animals (three seropositive and 206 seronegative decoys in the first sampling) could not be sampled in the second sampling. Results for HI were similar to those obtained in the first sampling, with 28 birds serologically positive to H5 (1.4%; 28/2006) and seven to H7 (0.3%; 7/2006). However, 66 decoys positive in the first sampling showed negative results in the second sampling, while 57 individuals seroconverted after the hunting period (individual incidence of 3.4%); 12 of them were confirmed as positive against H5 and three against H7 by HI.

The flock prevalence after the hunting period (65.2%; 88/135) was very similar to that found in the first sampling (Table 1). However, while the prevalence of H5-positive flocks slightly increased (to 11.8%; 16/135), the prevalence of H7-positive flocks slightly decreased (to 4.4%; 6/135) in the second sampling. Three flocks seroconverted to H5, two of them with only one positive birds detected and the other one with five new seropositive individuals. In addition, a flock seroconverted to H7 after the hunting period. On the other hand, three flocks positive to H5 and two positive to H7 in the first sampling, were negative in the second sampling.

AIVs RNA was not detected in any of the 47 cloacal swabs analyzed by RRT-PCR, including three animals positive against H5.

## Discussion

Even though wild and domestic birds have been the main target of surveillance programs and AI investigations, studies on the prevalence of AIVs birds reared in backyard are very limited in Europe [24] and, to the best of our knowledge; this is the first study on AIVs carried out in waterfowl used as decoys for hunting. The results indicate an enzootic circulation of AIVs in decoys in Southern Spain. Recent studies indicate that previous exposure to LPAIV confer some cross-protection, increasing the bird's probability of surviving HPAIV H5N1infection, and theoretically, these surviving birds could contribute to the spread of the disease [8], [13], [25]. On the other hand, experimental studies have also shown that development of LPAIV antibodies can result in a reduced magnitude and duration of shedding when infected with other AIVs including HPAIV H5N1 [10], [14], [25], thereby decreasing the likelihood of further transmission.

Active circulation of AIVs has been previously detected in wild and domestic waterfowl in Spain (Table 3) [15], [16], [11], [26]–[28], and highlights the role of these species in the epidemiology of AIVs [29], [30]. Higher seropositivity was previously found in both wild (33%; 306/927) and domestic (40%; 131/331) birds from different regions of Andalusia [15]. In contrast, a lower overall seroprevalence (6.2%; 44/712) was detected in wild waterfowl from the Doñana National Park (southwestern Spain) [16]. Recently, the AIVs prevalence found in central and northeastern Spain from faeces and tracheal swab samples were 2.6% (37/1435) and 4.5% (62/1374), respectively [26], [27] by means of rRT-PCR. A low prevalence (1.7%; 78/4578) was also found from fresh faeces in different Spanish wetlands using the same direct method [28]. Wide variations in prevalence of AIVs in wild birds have been also reported in other European countries (Table 3) [18], [31]–[39], [40]–[59]. The differences among studies could be due to the diagnosis methods, species analyzed, sample size, type of samples or environmental factors. The higher values obtained in the present study are logical considering that the technique used was an indirect method which detects antibodies against the nucleoprotein of AIVs type A. Detectable levels of antibodies against AIVs appear one to two weeks after infection and can last for several months, which also allowed the detection of birds that were infected prior to the sampling period. Owing to the intermittent viral excretion, direct diagnostic methods may result in the underestimation of AIVs prevalence [60]. In fact, faecal shedding of AIVs was not found in any of the 44 seropositive animals examined in the present study. Taking into account that fecal shedding of the virus is in general of less than 11–15 days [60]–[62], the possibility of detecting the virus was low. Our results are consistent with previous studies and suggest that surveillance for virus shedding alone may provide incomplete information on the transmission potential relative to surveys which also include detection of antibodies [25], [63]. Indirect techniques such as ELISA have potentially valuable applications for AIVs monitoring in bird species and should be considered when deciphering patterns of exposure, differential infection, and rates of AIVs transmission [25], [64].

**Table 3:** Prevalence of AIVs in wild birds in Europe.

Location	N	POSITIVE (%)	Type of test	Reference
Spain	208	43	ELISA/IH	Arenas et al., 1990
Spain	712	6.2	ELISA	Astorga et al., 1993
Spain	3500	8	RRT-PCR	Barral et al., 2008
Spain	686	7.9	RRT-PCR	Busquets et al., 2010
Spain	628	4.6	RT-PCR	Pérez-Ramírez et al., 2010
Spain	4572	1.7	RRT-PCR	Pérez-Ramírez et al., 2012
Portugal	3561	2.3	RT-PCR	Henriques et al., 2011
Portugal	1542	4.5	RRT-PCR	Tolf et al., 2012
Italy	1039	52.2	ELISA/IH	De Marco et al., 2004
Italy	326	66	ELISA/IH	De Marco et al., 2005
Italy	3000	5	RRT-PCR	Cattoli et al., 2007
Italy	4083	8	RRT-PCR	Terregino et al., 2007
Italy	147	0/1,3	RT-PCR/ELISA	Delogu et al., 2012
Italy	2023	2.2	RT-PCR	Kelvin et al., 2012
France	799	6.9	RT-PCR	Lebarbenchon et al., 2009
France	2901	5.4	RT-PCR	Lebarbenchon et al., 2010
France	205	15	RT-PCR	Vittecoq et al., 2012
Belgium	7500	0.02	RT-PCR	Marché et al., 2013
Germany	5864	3.7	RRT-PCR	Rabl et al., 2009
Germany	1402	1.07	RRT-PCR	Pannwitz et al., 2009
Germany	12652	2.3	RRT-PCR	Lang et al., 2010
Netherlands	132	51.5	ELISA	Verhagen et al., 2012
Denmark	1381	3.1	RT-PCR	Bragstad et al., 2007
Switzerland	2000	4	RT-PCR	Baumer et al., 2010
Sweden	4800	12.5	RT-PCR	Wallensten et al., 2007
Sweden	7728	13.1	RRT-PCR	Latorre-Margalef et al., 2013
Norway	604	13.2	RT-PCR	Jonassen & Handeland, 2007
Norway	1529	12.5	RT-PCR	Germundsson et al., 2010
Norway	2417	15.5	RRT-PCR	Tønnessen et al., 2013
Austria	3151	3.77	RT-PCR	Fink et al., 2010
Georgia	8343	1.6	RT-PCR	Lewis et al., 2013
Turkey	402	0.49	RRT-PCR	Albayrak et al., 2010
Slovenia	2547	4.4	PCR	Slavec et al., 2012
Finland	310	1.6	ELISA	Lindh et al., 2008
Northern Europe	8500	1	RT-PCR	Fouchier et al., 2003
Northern Europe	36809	2.6	RT-PCR	Munster et al., 2007

Even though most of the decoy flocks analyzed (70.6%) were mixed, including geese and ducks, and individuals were bred with similar management conditions, a significantly higher seroprevalence was found in geese compared to ducks. Differences in the prevelence of AIVs among species have been previously described and could be associated to variations in the immunological response, behavior, density-related patterns or gregariousness patterns. In the majority of studies [26], [27], [30], [31], [33], [36], [59], the highest prevalences were detected in ducks. The dabbling behaviour of some ducks seems to play an important role in their higher prevalences as the AIVs excreted in faeces remain in surface waters and are ingested by other ducks, while geese graze in pastures and agricultural fields [59]. However, in North America and Alaska, among hunter-harvested species, the greater white-fronted geese (*Anser albifrons*) and Emperor geese (*Anser canagica*) had the highest prevalences, respectively [25], [65]. A higher susceptibility to AIVs infection was found in geese compared to swans [66], while ducks are considered more susceptible than chickens [67]. Additional studies would be needed to explain these differences in prevalence among species.

The flock seroprevalence levels indicate widespread circulation of AIVs in decoys in Andalusia. However, the results show that the spatial distribution of AIVs in decoys in Andalusia was not homogeneous. Statistically significant differences in seroprevalence were observed among municipalities, with highest seroprevalences in the regions located close to the river Guadalquivir (Cádiz and Seville), the largest river in Andalusia. The seropositivity obtained in Huelva (6.1%) was similar to that previously detected by Astorga et al. (1994) in Doñana National Park, the main wetland in Spain, and within the route of different migratory species between Europe and Africa. Environmental factors have been associated with the risk AIVs transmission [9], [68]. In Andalusia, 36 of the 41 (85.4%) hunting areas for aquatic birds (total area: 47986 ha) are located in the Seville province (42973ha), and a 93.7% of the 25720 birds hunted in 2012 were obtained in this province [69]. In this sense, the risk of contact between decoys and wild birds may increase in flocks close to wetlands, especially in decoy flocks reared outdoors.

The individual incidence between the first and the second sampling was 3.4%. A total of 57 individuals seroconverted, which confirms AIVs infections during the study period. The results may be associated to contact with infected decoys, migratory and resident infected wild birds or to the presence of AIVs in the environment where decoys are kept during the hunting activity [70]. The second sampling was carried out just after the winter period coinciding with the greatest presence of migratory birds in Spain. Studies carried out in this and other Mediterranean countries have shown that wintering grounds favor the introduction of new AIVs strains and their transmission among resident birds [27], [36], [71].

The detection of antibodies against H5 and H7 by HI indicates that birds were exposed and responded serologically to the contact with these AIVs subtypes. In fact, 15 out of the 57 seroconverted decoys analyzed in the second sampling were confirmed by HI as positive against H5 (12) and H7 (3), indicating recent infections with both subtypes. Although the pathogenicity of the circulating strains could not be determined, the results indicate that decoys could represent a risk for HPAIVs emergence associated to genetic mutations, as has been previously demonstrated [30]. The fact that H5 and H7 are circulating in the area, may result in the introduction of these subtypes into poultry and the development of LPAIV or even HPAIV outbreaks, as the introduction of AIVs into poultry are primarily the result of wild bird activity, not only as a consequence of direct contact, but also indirectly via contaminated water [9]. The seropositivities to H5 and H7 subtypes found are in accordance with those previously reported in central and northern Spain by RRT-PCR [11], [26], [27], which suggests a limited circulation of both subtypes in Spain. Limited H5 and H7 subtypes circulation have been also found in other European countries [18], [34], [55], [72]. LPAIVs circulation has been frequently detected in the Iberian Peninsula, being the H3N8, the H4N6 and the H1N1 the most common subtypes detected in Spain [26], [28], and the H10N7, the H9N2 and the H11N3 in Portugal [32].

In conclusion, the results of the present study confirm a widespread circulation of AIVs in waterfowl species used as decoys in Andalusia. The seroprevalence obtained indicates that decoys are frequently exposed to AIVs and may potentially be useful as sentinels for AIVs monitoring. The seropositivity against AIVs H5 and H7, suggest that decoys can act as reservoirs of these subtypes, which may be of animal and public health concern. Because of the direct contact among decoys, wildlife and human, these species should be considered as risk species for the transmission of bird-borne pathogens, including AIVs. Control measures to limit transmission from wild birds to decoys as well as from decoys to wild birds should be implemented.
